# “When a dog bites someone”: Community and service provider dynamics influencing access to integrated bite case management in Chad

**DOI:** 10.3389/fvets.2022.866106

**Published:** 2022-10-10

**Authors:** Alladoumngar Madjadinan, Nodjimbadem Mbaipago, Ndèye Marème Sougou, Mayassine Diongue, Jakob Zinsstag, Kathrin Heitz-Tokpa, Monique Lechenne

**Affiliations:** ^1^Centre de Support en Santé Internationale, N'Djamena, Chad; ^2^Institute of Health and Development, Dakar, Senegal; ^3^Public Health Department, Université Cheik Anta Diop de Dakar, Dakar, Senegal; ^4^Department of Epidemiology and Public Health, Swiss Tropical and Public Health Institute, Basel, Switzerland; ^5^University of Basel, Basel, Switzerland; ^6^Centre Suisse de Recherches Scientifiques, Abidjan, Côte d'Ivoire

**Keywords:** rabies, Chad, One Health, PEP access, integrated bite case management (or alternatively IBCM)

## Abstract

This study aims to identify factors on the community, the human health and the animal health provider level that determine access to Post Exposure Prophylaxis (PEP) and animal rabies diagnosis in the light of a future integrated bite case management (IBCM) approach for rabies control in Chad. The study was embedded in an overall project conducted from 2016 to 2018, to determine rabies burden and vaccine demand in West and Central Africa. Data collection took place during the projects closing workshops with stakeholders organized between August and September 2018 in the three study zones in Chad covering Logone Occidental and Ouaddaï province and parts of Hadjer Lamis and Chari Baguirmi province. A qualitative approach based on focus group discussion and in-depth interviews was used to get insights on access to care and animal investigation after suspected rabies exposure. A total of 96 participants, including 39 from the community (bite victims, dog owners) and 57 human and animal health providers (health center managers, chief veterinary officers, chief district medical officers, chiefs of livestock sectors) contributed to the study. Based on an existing conceptual framework of access to health care, several points of dissatisfaction were identified, in particular the unaffordability of human rabies vaccine for PEP (affordability) and the distance to travel to a health facility in case of a bite (accessibility). In addition, there are unfavorable attitudes observed highlighted by the importance given to traditional or local rabies care practices to the detriment of PEP (acceptability) and a low level of knowledge among Chadian communities regarding bite prevention, coupled with a very inadequate information and awareness system regarding the disease (adequacy). As for human and veterinary health services, both sectors suffer from insufficient resources for PEP on the human health and rabies diagnosis on the veterinary side impacting negatively on availability and accessibility of both these services. Action to improving provision of rabies health services and increasing knowledge about risk and prevention of the disease among the population need to be undertaken to implement IBCM, improve access to PEP and achieve the goal of eliminating dog mediated human rabies by 2030 in Chad.

## Introduction

**Figure 1:**
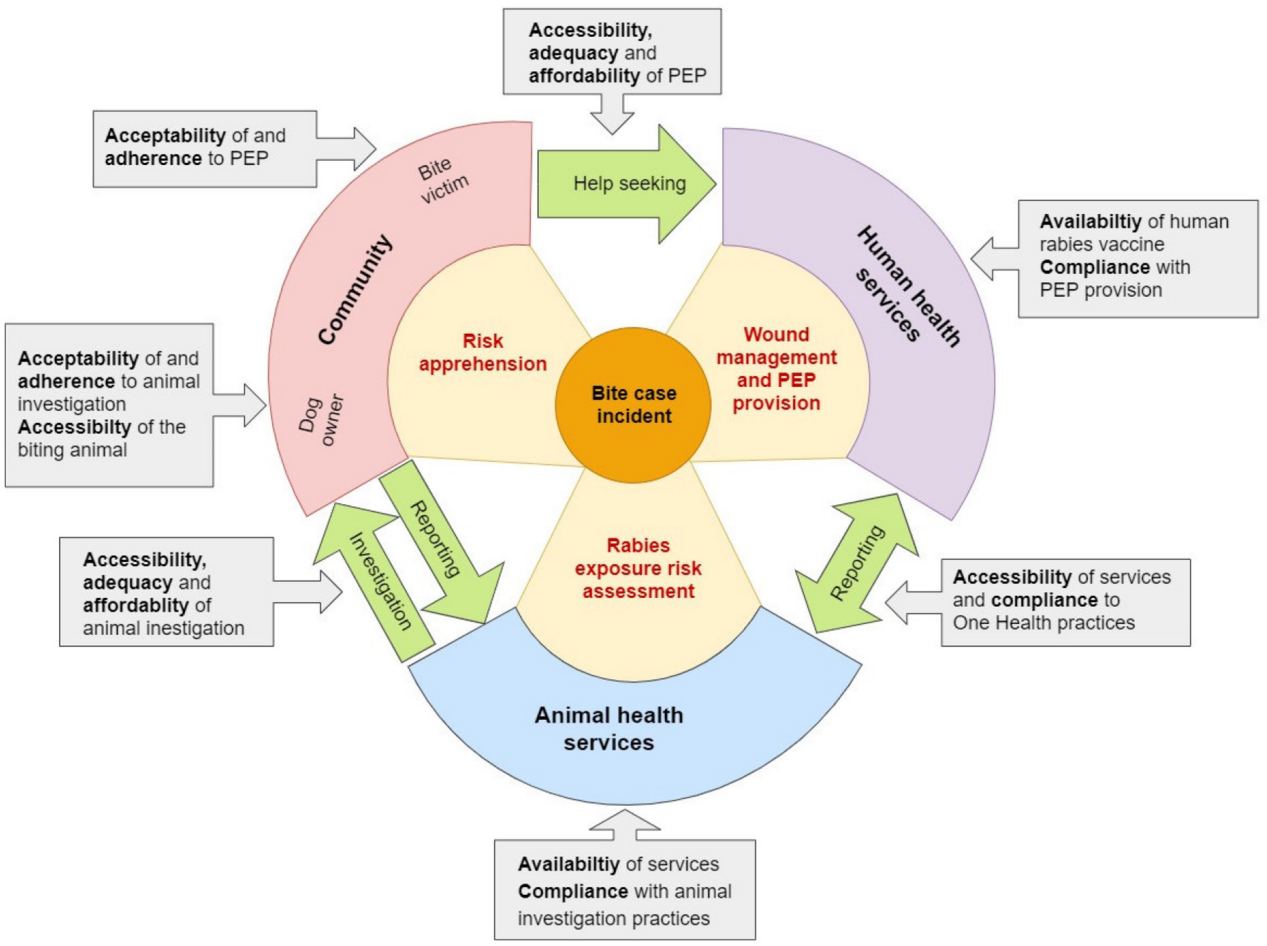
The integrated bite case management (IBCM) access cycle with the various access elements described by Obrist et al. (15) (gray boxes) influencing on different levels of the cycle (community, human health and animal health services). Red text in the cones describes the roles of the primary actors involved in IBCM. Green arrows highlight the communication actions that need to be undertaken between the three levels.

Rabies is a zoonotic disease caused by an RNA virus of the genus Lyssavirus and transmitted through the bite of an infected animal (1). It is a fatal disease once clinical symptoms appear (2). Despite the fact that effective tools for controlling this disease are known, including canine and human vaccination, more than 59,000 people die from rabies each year worldwide (3). The domestic dog is the main source of human exposure and the primary reservoir of rabies in developing countries (4). Post-exposure prophylaxis post-exposure prophylaxis (PEP) is the only way to avoid rabies after exposure, but often PEP remains difficult to access in low-income countries (5). A recent study revealed that improved access to PEP, potentially supported by free provision of human vaccine by GAVI, the vaccine alliance, could prevent more than 400,000 deaths in a timeframe of 15 years (6). Together with large-scale dog vaccination interventions, dog mediated human rabies cases could be eliminated in the near future as outlined in a global strategic plan by the tripartite [WHO, FAO, OMSA (former OIE)] and the Global Alliance for Rabies Control (GARC) (7). PEP and dog vaccination combined are highly cost-effective interventions, provided that human health and veterinary services collaborate well together (8). Investigation of the rabies suspicion status of a biting animal through an animal health professional can help to assess a patient's exposure risk and thus ensure adequate use of human rabies vaccine that is currently in short supply (9, 10). This risk assessment and advanced surveillance based on a One Health (OH) approach is called integrated bite case management (IBCM) (11) and has been proven to be effective in various settings including Haiti (12), Tanzania (13) and the Philippines (14). To describe effectiveness of IBCM the elements of access to health care described in the framework proposed by Obrist et al. (15) can be applied on community, human health and veterinary health levels, respectively. These seven elements are: (1) Availability: The existing health services and goods meet clients' needs; (2) Accessibility: The location of supply is in line with the location of clients; (3) Affordability: The prices of services fit the clients' income and ability to pay; (4) Adequacy: The organization of health care meets the clients' expectations; (5) Acceptability: The characteristics of providers match with those of the clients; (6) Compliance: health care providers adhere to best practices; (7) Adherence: clients adhere to recommendations. Figure 1 illustrates these elements in the context of IBCM.

In Chad, rabies is endemic, and despite numerous studies carried out in this country in recent years, control measures have not evolved. The Chadian dog population is highly dynamic and mostly free roaming (16). Nearly half of all households (45%) own dogs and the dog to human ratio is 1:8 (17). Less than 2% of the Chadian dog population is vaccinated and access to PEP for dog bite victims is as low as 8.5% (18). The population of Chad is confronted with problems of inadequate human and veterinary health structures. There are recurrent challenges of insufficient human and veterinary health personnel as well as difficulties in accessing health care in general and rabies prevention in particular (19). It has to be noted that PEP in Chad includes only active vaccination, passive vaccination with rabies immunoglobulin (RIG) that according to WHO recommendations would need to be applied for all category 3 exposures (11) is virtually not available in Chad.

From 2016 to 2018, a project to estimate the burden of rabies and PEP demand in West and Central Africa was conducted with financial support from GAVI (20). In Chad, this funding has enabled the extension of canine rabies surveillance to four administrative provinces through establishment of decentralized diagnostic units (21), collection of data on bite exposure on household level and access to PEP on health facility level (18). The results of the household survey showed that the incidence of dog bites ranged from 2.1 to 9.5 per 1,000 people per year depending on the region, and almost half of the bite victims were under 15 years of age (18). However, due to many reasons including socio-economic, geographical and cultural factors, only 33% of households seek care in medical facilities in case of a bite (18). Traditional care is seen to be practiced widely and traditional healers were consulted more often than veterinary services (22). Assessments of knowledge, attitudes and practices of health and veterinary workers also revealed numerous problems in the organization of care for people exposed to rabies (23, 24).

With regard to rabies service delivery, human and veterinary health personnel were not used to collaborate for the management of bite victims, prior to the GAVI project. During the project a OH approach was piloted in the study regions with the aim to improve communication between the human and animal health sector (20). Despite this effort, among overall 1,540 bite cases reported during the health facility survey, only 20% had been the subject of effective collaboration between human and veterinary health services (18), which illustrates the persistence of communication difficulties between the two sectors even after reinforcement through an OH project.

The findings from the various quantitative studies of the GAVI project allowed us to assess rabies risk and the need for PEP on the national level and to identify a lack of access to appropriate health and veterinary facilities with well-trained health and veterinary workers (18, 21, 23). However, the purely quantitative data did not allow us a deeper understanding of the mechanism of PEP access and implementation of joint case management by human and veterinary health services. Therefore, we identified the need to complement the quantitative data with a qualitative data collection to better explain the barriers to seeking rabies care. The aim of this qualitative study presented here is to identify factors affecting access to IBCM in Chad on community and service provider level across the various elements of the health care access framework described above (15). The combined approach for data collection on both the demand (community) and the provider side (animal and human health institutions) helps to understand the dynamics between the two and identify points of leverage to improve the access cycle (Figure 1). Together with the already published results of the quantitative studies, the findings of this complementary qualitative study will help to plan future rabies control activities in Chad, in particular implementation of IBCM in public veterinary and human health facilities and well-targeted public awareness campaigns.

## Methodology

### Study background and aim

The qualitative study presented in this paper is part of a large-scale research project implemented in Chad, Côte d'Ivoire and Mali, to estimate the burden of rabies and vaccine demand in West and Central Africa, funded by GAVI described in Léchenne et al. (20). In Chad, this research project lasted from January 2016 to September 2018 and was implemented in three study areas belonging to four administrative provinces (see chapter Study areas below). As described in the introduction the project included quantitative data collection on household level (bite case survey) (18), health facility level (bite case reporting and PEP follow-up) (18) and veterinary facility level (animal rabies reporting and diagnosis) (21). The qualitative study presented here was initiated to complement these quantitative studies. The data collection was conducted from August to September 2018 on the occasion of the GAVI project closing workshops in the selected provinces. Data was collected through Focus Group Discussions (FGD) and in-depth interviews (IDI) with community representatives and representatives of the institutional level (see chapter Selection of study participants below). The objectif was to get a parallel insight from the community and the care provider level on the factors influencing access to PEP and implementation of IBCM in both these backgrounds. We chose this approach because access to PEP on the human side and appropriate rabies investigation on the animal side is shaped by the balance between availability and demand. If demand for a particular service is low, this service becomes unprofitable for a provider and is therefore not supported and vice versa. Therefore, we understand the mechanism of PEP access as a circular process that can only be understood through a holistic view integrating all concerned levels of this mechanism. Presenting findings from the demand side (community level) and the service provision side (institutional level) together, helps to identify negative or positive dynamics influencing the access cycle (Figure 1).

### Study areas

The study areas were the same than those chosen for the overall GAVI-project: The Ouaddaï province (OU), the Logone Occidental province (LO) and the rural area around N'Djamena within a radius of 100 km around the city covering parts of the province Chari Baguirmi (CB) and Hadjer Lamis (HL). These four areas were selected to cover different demographic characteristics of the country. According to data from the 2009 general population and housing census (25), Ouaddaï has a population of 997,257 and is a predominantly Muslim region, with the largest ethnic groups being the Maba and Massalit. Ouaddaï comprises four health districts: Abéché (urban center); Adré, Abdi and Abougoudam (rural districts). The Logone Occidental region has a population of 961,503, the main ethnic groups are the Ngambaye and Christianity is the dominant religion (25). This province is also divided into four health districts which are: Moundou (urban center); Beinamar, Laoukassy and Bénoye (rural districts). The third study area of rural N'Djamena is covering two health districts of the Chari-Baguirmi province (Mandélia and Dourbali) and two health districts of the Hadjer-Lamis province (Massaguet, Mani). The two district cover about 700,000 inhabitants or 50% of the population of the two provinces combined (25). In these regions live the Baguirmien, the Arabs, the Barma and the Kotoko, and the Sara-Ngambaye ethnic groups. Each study area covered four health districts.

### Selection of study participants

Based on our experience from the quantitative data collection within the GAVI-project and our research aim, we identified six stakeholder groups from which to collect in-depth qualitative data: bite victims (BV), dog owners (DO), health center managers (HCM) and chief veterinary officers (CVO), chief district medical officers (CDMO) or district hospital chiefs (DHC) and chiefs of livestock sectors (CLS). Drawing on respondents from the quantitative survey, sampling was carried out in a purposive manner to include informants from the different groups identified and from all health district of the study areas. The following sampling strategy was used in order to cover a wide spectrum/range of situations encountered in the quantitative study.

- A selection of four dog owners by study area (1 per health district) having reported a bite incident during the extended animal surveillance period (21); two residing near (<5 km) and two residing far from a veterinary station (>5 km).
- A selection of four bite victims by study area (1 per health district) encountered during the health facility survey (18); two residing near (<5 km) and two residing far from a health center or hospital (>5 km).
- Two health center managers by health district in all study areas, amongst them one manager whose facility had recorded many (>10) and one whose facility had recorded very few bite victims (<10) during the health facility survey (18).
- Two chief veterinary officers by health district in all study areas, amongst them one whose facility had recorded many investigated dog bite cases (>10) and one whose facility had recorded very few investigated dog bite cases (<10) during the extended animal surveillance period (21).
- All chief district medial officers of the health districts covered by the GAVI-project or in case of absence the district hospital chief.
- All chief of livestock sectors corresponding to the health district zones covered by the GAVI-project.

The recruitment of participants was done with the help of the quantitative data basis (health facility survey and animal case reporting) and according to the inclusion criteria mentioned above. Identified participants were contacted by phone or through the help of the respective local project collaborators (veterinary officers and health center managers). They were informed of the purpose of the study in order to obtain their free consent to participate. Upon agreeing, they were informed of the date and place of the IDI and FGDs. Data was collected in three major cities: Abéché, N'Djaména and Moundou. The timing of the data collection was coupled with the 2-day closing workshops of the GAVI-project held in these three cities between August and September 2018. Since the window of opportunity for the data collection was very narrow, participants that were not available on short notice on the respective dates for either the FGD or the IDI in each area could not be replaced. This led to overall fewer study participants than initially planned (Table 1). Participants from the surrounding villages and provinces received 10,000 CFA francs each as an incentive for transport, while those living in the respective city where the workshops took place received 5,000 CFA francs.

**Table 1:** Description of participants by stakeholder group and data collection tool.

	**In-depth interviews**				**Focus group discussion DO and BV**		**Focus group discussion HCM and CVO**		**Focus Group Discussion CDMO/DHC and CLS**	
**Study areas**	**DO**	**BV**	**HCM**	**CVO**	**No. FGD**	**No. participants**	**No. FGD**	**No. participants**	**No. FGD**	**No. participants**
Ouaddaï	2/4	1/4	1/4	1/4	1/1	8/8	2/2	14/16	1/1	8/8
Logone Occidental	3/4	3/4	1/4	1/4	1/1	6/8	2/2	12/16	1/1	6/8
Hadjer Lamis and Chari Baguirmi	4/4	4/4	0/4	0/4	1/1	8/8	1/2	07/16	1/1	6/8
Total	9/12	8/12	2/12	2/12	3/3	22/24	5/6	33/48	3/3	20/24

Both target numbers and the actual achieved numbers are provided by group and tool (achieved/targeted).

### Collection tools and methods

FGDs were used to understand collective perceptions and attitudes toward bite case management and PEP seeking, while IDI were needed to understand the personal motivations behind health-seeking and reporting (for bite victims and dog owners) or service provision (for participants of health and veterinary services). The interview and focus group guide for victims and dog owners was structured along the following axes: knowledge about rabies, attitudes in case of a bite and obstacles to obtaining appropriate treatment. The interview and focus group guide for health and veterinary services was structured around the following main themes: quality of the services currently offered, demand of these services by the population, needs of the services for effective rabies control, perception of the OH approach. The focus group and interview guides by participants group can be found in the Supplementary material S1.

Field data collection was entirely carried out by the student in charge of the research (lead author), assisted by a student from the Afrique One-ASPIRE consortium, in Master's degree at the University of Korhogo in Côte d'Ivoire (second author). The senior coordinator of the GAVI study (last author) supervised data collection. All IDI and FGDs were recorded by a Dictaphone and with the prior oral consent of the respondents. Data collection was conducted in French and sometimes in local languages.

For the FGDs, participants were separated into community level (DO and BV), the primary health proivders (HCR and CVO) and the administrative level (CDMO, DHC and CLS). FGDs took place in a quiet location on the premises of the workshop location. Group sizes ranged from min six to maximum eight participants depending on the actual presence of invited people. Table 1 depicts the number of participants by stakeholder group and data collection tool and also provides achieved vs. targeted numbers. On the community side, nine IDI with dog owners, eight IDI with bite victims and three mixed FGDs were conducted. Concerning the institutional side, four IDI (two with HCR and two with CVO) were conducted. Unfortunately, in the third study area no IDI for this stakeholder group could be done. However, participation of HCR and CVO in FGD was good with overall five mixed FGDs conducted and a total of 33 participants. With CDMO, DHC and CLS only FGD were conducted. Since the targeted number was fewer than for HCR and CVO only one FGD per study area took place.

The fact that the initial targeted number of participants was not reached has to do with challenges linked to mobility during the rainy season. At the time of data collection in August, not all participants were able to attend the data collection location at the district capital. However, data saturation was reached before the initial study size was obtained allowing for a comprehensive analysis.

### Data analysis

Translations into French were double checked by both research facilitators. The transcribed texts were imported into the Nvivo12 software version, and after several readings, the text corpus was coded according to the themes of interest in the study. Taking into account the themes addressed, the main information on the socio-cultural and organizational factors that influenced the perceptions of the populations and providers for the control of rabies through the OH approach, was analyzed with the content analysis method. The content analysis method followed an inductive approach where the main themes identified were derived from the data. The analysis was performed by the first and second authors of this manuscript. They both have acquired ample experience in rabies research during several former projects in Chad and were members of the local GAVI project team. Translations of quotes into English were slightly edited to increase readability.

### Ethical aspects

Ethical approval for the overall study, covering research activities in the three GAVI-project countries (Mali, Côte d'Ivoire and Chad), was granted by the Ethics Committee of Northern and Central Switzerland (EKNZ). The specific research activities of the GAVI-project in Chad received research approval from the Chadian Ministry of Public Health (MSP) (No. 1569 / MSP / SE / SG / DGAS / 2016) and the National Bioethics Committee of the Chadian Ministry of Higher Education (No. 298 / PR / MESRS / SG / CNBT / 2016). Oral consent was obtained from each participant before data collection.

## Results

### Characteristics of the participants

On the community level, 39 dog owners and bite victims participated in the study, 31% (12) in Logone Occidental, 28% (11) in Ouaddaï and 41% (16) in the study area around N'Djamena. There were more men 82% (32) than women 18% (7). Of the participants 67% (26) were Christians and 33% (13) were Muslims. The majority were engaged in agriculture and livestock rearing 67% (26), 21% (8) were wage earners, 8% (3) were traders and 4% (2) were students. Regarding the level of education of the participants 49% (19) had no formal education, 12% (5) had primary education, 26% (10) had secondary education and 13% (5) had higher education.

On the institutional level, 57 providers participated in the study, 35% (20) in Logone Occidental, 42% (24) in Ouaddaï and 23% (13) in rural N'Djamena. There were 84% (48) from the human health and 16% (9) from the veterinary health sector. The age of the respondents ranged from 35 to 62 years, with an average age of 47 years, and there were 55 men and two women. The respondents were composed of 53% (30) nurses, 22% (12) doctors, including two veterinarians, the other 25% were heads of veterinary posts (usually livestock technicians) (4), senior livestock technicians (3), technical health officers (4), senior health technicians (3) and one pharmacist.

### Access determinants on community level

#### Knowledge of the population about rabies

Participants in the FGDs and IDI described rabies as a dog disease transmitted to other dogs or to humans by bite. The signs commonly cited for animals were fury, aggressiveness and altered behavior; as for human rabies, participants indicated that rabid people barked like dogs.

Q1: “*When I was in Moundou, there were many cases. All the rabid dogs, when they bite another dog there, some week later the dog there also becomes rabid. It's a disease that attacks the dog. It becomes furious, its eyes become red and it becomes aggressive. It doesn't even recognise its owner. Everything it comes across, it will bite, and when it bites a person, the disease will develop in the person who will perhaps end up dying. But I was a kid when I saw the rabies victims taken to hospital. I was told that they screamed like dogs, too, but I didn't see that. But I know that rabies is a disease that kills*.” (IDI: DO, male, CB)

Q2: “*Before it disappeared, it didn't have this behaviour. But when it was bitten by another dog and it disappeared for 11 days, as soon as it came back it changed. It doesn't recognise anyone, it bites everything, even the wood. That's why we had to kill it*.” (IDI: DO, male, LO)

Some participants incorrectly stated that the disease that the dog transmitted to humans was tuberculosis. The same participant believed that a deranged mind or madness was the result of rabies transmission.

Q3: “*Some people say that a dog can also transmit tuberculosis to a man. Also a deranged mind, they say that it is the dog that gives that*.” (IDI: BV, male, HL)

Overall, the rabies knowledge of the participants from the community side seems to be quite good, but this has to be interpreted with care, since all participants had prior experience with a bite or a rabies case and were in contact with the GAVI project that included awareness campaigns. Accounts from the care provider side showed that lack of knowledge in the community, especially about rabies risk even in case of a minor wound, is hindering access:

Q4: “*Many people do not come to the health services to ask for the vaccine. I have had enough cases in my area. People have died from rabies, because there are some people who are bitten by the dog and indeed the dog is rabid. They treat themselves at home with traditional care. Afterwards, the wound is healed, and they think they are cured because the wound is healed. They don't know that there is the virus in the blood and after some time, […] as soon as the virus attacks the brain, they come and get to us, that such and such a day our relative was bitten by a dog and by then it is already late. They can't understand that this is what causes a lot of health problems*.” (Extract from FGD: CVO, male, LO)

#### Attitudes and practices of the community

When asked about practices in the event of exposure to bites, participants report positive actions such as the washing of the wound, recourse to medical structures to receive treatment and the need to put the animal under observation. However, the FGDs showed that the majority of respondents are not particularly aware of any mode of rabies prevention or post-bite prophylaxis. They simply adhered to the principle of going to a medical facility to seek treatment after exposure instead of consulting traditional healers. Some respondents were aware of the need to take a biting animal to the veterinary station to ascertain whether it is ill with rabies as opposed to killing it immediately.

Q5: “*When the child was bitten, we washed the wound and also took the child to the hospital, he received injections. And the dog we took it to the veterinarian and the head of the post to examine the dog.”* (Extract from FGD: BV, male, CB)

Q6: “*It's the medical treatment, which allows us to save many people bitten by rabid dogs. But for a person bitten by a rabid dog to be cured by traditional treatment, I have never seen that*.” (Extract from FGD: BV, male, LO)

On the other hand, there are also people who do nothing at all after a bite case, either through ignorance or negligence. This is what this victim of a bite from DS Laokassy tried to explain in his words:

Q7: “*She was a woman, the dog bit her but they did not get her the rabies vaccine and they did not take her to the hospital either, until the rabies broke out. That is when they brought her in and then the doctors could not save her. She is a person but she barks exactly like a dog like that, that's when I understood that it's rabies, apparently the virus has reached her whole body*.” (IDI: BV, male, LO)

The interviews also report the refusal of a victim's father to respect the referral instructions of the head nurse of the health center, which eventually led to onset of symptoms some weeks later and eventually the child's death. This refusal could be both a result of negligence and difficulties to travel the long distance to the facility with vaccine in stock.

Q8: “*We have a child from the village who was bitten by a rabid dog. His father brought him to the Djarmaya Health Centre, the RCS (Responsible of the health center) transferred him to the Massaguet District, so that he could receive the vaccine. But the father said we want injections [against tetanus] and we are going to return to the village. Some 40 days later the child started to get sick, when he was given water, he said: ‘No, I don't want to!' He refused to drink and he died*.” (IDI: BV, male, HL)

Because all participants from the community had sought help after a bite incident either at a human health or at a veterinary facility, attitudes and practices reported by them were mostly positive. However, the FGDs and IDIs on the care provider level revealed certain barriers related to attitudes and practices of the community. For example, instead of seeking help in a health facility and get PEP, some patients find the alternative or have a preference for traditional treatments. Providers report that traditional treatment is detrimental to the health of the population, especially because most of these traditional practices prohibit patients from seeking other treatment, especially bio-medical care.

Q9: “*Whenever there is a bite case, if it is a child, the parents refuse, they oppose medical treatment. They say that they have already given traditional treatments and that the victim should not take even one tablet of Paracetamol. As soon as he takes it, he will die and we are obliged to accept it*.” (Extract from FGD: CVO, female, LO)

The health service providers felt that the reasons for using traditional health services are multiple: They are linked to the poverty of local communities (see section on affordability and geographical accessibility below), but interviews also reveal that socio-cultural constraints built around beliefs are a strong factor for taking resort in traditional alternatives. Some even see the spirit of witchcraft in rabies, which reinforces the belief that it should be cured by a traditional healer.

Q10: “*There are many reasons for this [to consult a traditional healer], sometimes it is an economic problem, when the patients do not have the means to pay, so they take resort in traditional care. But sometimes it's a socio-cultural constraint, people give much more importance to traditional treatment than to modern treatment, especially in Beinamar. So sometimes we try traditional treatments and when it doesn't work, we go to the hospital. Sometimes, in the case of rabies, they think that a witch doctor has taken the person and that makes them reluctant [to consult a health centre]*.” (Extract from the FGD: CDMO, male, LO)

Cultural and religious practices also influence access of dogs to veterinary observation and rabies diagnosis. For example, a relevant obstacle in some Muslim communities is the religious taboo of handling dogs and especially dog carcasses. On the other hand, the cultural practice of eating dogs in some Christian and Animist communities has a negative impact on animal rabies surveillance. Accounts from health providers show that killing and eating of a dog that has bitten someone is widely practiced.

Q11: “*If people know that you have cut the head off a dog they will not eat with you*.” (Extract from FGD: CVO, male, OU)

Q12: *When a dog bites someone, we want to put the dog under observation and if it dies, the analysis must be done at this level so that we understand. But people kill the dog and they eat up to the head, it is difficult*.” (Extract from FGD: HCM, male, LO)

According to both the population and service providers, there is a need to diversify sources of information and awareness, to inform and influence individual and community habits on rabies knowledge, attitudes and practices. Especially in a context where a large number of the population lives in rural areas, information carried by public media does not always reach remote villages.

Q13: “*Because if the vaccine is available but the community doesn't know about it, it would be difficult. So maybe we need to inform more at the peripheral level. As for the rabies vaccine, when a dog bites someone, you don't automatically have to kill the dog and eat it. So you have to make people aware of this.”* (Extract from FGD: HCM, male, LO)

#### Affordability and geographical accessibility

In the opinion of the people interviewed, many people cannot seek care or follow treatment when they were bitten because of their socio-economic conditions. Most of the respondents said that human rabies vaccines are very expensive and thus virtually unaffordable to them, for example one would have to sell an asset such as an ox to get it.

Q14: “*We want the human rabies vaccine to always be available in hospitals, because before there was no vaccine and if you could find it, it's expensive. Once, a bite victim travelled to Koumra (about 200 km away) to get a dose for 12,000 FCFA [about 20 USD]. So to treat a person bitten by a rabid dog, you either have to sell an ox or some other good to save that person*.” (Extract from FGD: BV, male, LO)

The population's view of the high cost of the vaccine for PEP is also echoed by health service providers.

Q15: “*The cost of the vaccine here is extremely expensive. […] Common people cannot spend more than 50,000 for one child and if he has three children, where is he going to find the money*.” (Extract From FGD: HCM, male, CB)

The lack of financial means and thus the unaffordability of PEP, forces certain segments of the population to fall back on traditional care as described above.

Q16: “*It is poverty that leads people to give importance to traditional treatment, which prevents them from going to the health center*.” (IDI: HCM, male, LO)

A district medical officer explains that the government is well aware of this problem and that there is the willingness to provide free PEP on the institutional level.

Q17: “*In fact, in human health, well before this project, the Chadian state had planned something for the care of people bitten by animals suspected of having rabies. Thus, free care was even instituted for these people. So, a certain amount of anti-rabies medication was made available to the health facilities, […]. With the project, it is true that it allowed us to intensify the action and even if the project has come to an end, we are going to take up the results of this project to be able to do better in the future*.” (Extract from FGD: CDMO, male, OU)

In all three study areas, participants reported PEP access difficulties related to geographical barriers. Distance and transport difficulties is presented by the respondents as a factor that discourages patients from seeking post-bite treatment.

Q18: “*To go and get this anti-rabies treatment, you need a means of transport, and maybe that's it. But even if you don't have transport you have to ask your parents, they don't have enough money either*.” (Extract from FGD: BV, male, LO)

The difficulties related to distance are also reported by the health providers. The results from the FGD and IDI among care providers revealed in fact that distance is affecting proper health seeking of the community in the first place, but also on adherence to treatment. According to health personnel, the long distances are a reason for people to prefer traditional care, especially for wounds considered not very severe. Even if they seek help, some patients only take two or three doses of vaccine and do not complete PEP, because of the distance. There are also patients who cannot seek help during the rainy season because some areas become inaccessible.

Q19: “*For the bitten person to be able to travel 180 km to come and get the vaccine in the district poses a problem, because there is a barrier to transport and people are very reluctant to travel. Because for [the patient], it is not serious, he prefers to go and see the traditional healer and it is when it is bad that they come for the first time*.” (Extract from FGD: CDMO, male, OU)

Q20: “*There are some patients who abandon the treatment, because they travel long distances to come and when they take [it] twice, they think that the person is already saved, and they don't comeback anymore.”* (IDI: HCM, male, LO)

### Access factors on the institutional level

#### Adequacy of human and animal health services concerning rabies

Under the terms of law 09/PR of 19/05/2004, in its article 5, rabies was included in the list of notifiable infectious diseases subject to compulsory declaration throughout the territory of the Republic of Chad, but this law has not been reinforced and followed up. Rabies surveillance is currently neglected by both the human health and the livestock sector and the collection of data on animal rabies is therefore challenging. Veterinary health service providers also believe that it is due to the lack of knowledge of this law that the population is not reporting suspect cases.

Q21: “*We have intervened in diseases that are legally notifiable, including rabies. But in livestock surveillance rabies was not included. Here, we are too interested in the economy, hence in animals that can be profitable, which is why rabies is neglected in surveillance*.” (Extract From FGD: CLS, male, LO

Q22: “*At the level of the Ministry of Livestock, we have lists of diseases. Rabies is one of the diseases considered contagious and that we must declare a case of rabies, [as stipulated by the] law 09 of May 2004. So for me, it is the lack of knowledge of the population that does not allow to pass on the information*.” (Extract from FGD: CLS, male, LO)

The preference of animal health services to deal with economically more profitable diseases and species is echoed by responses received by dog owners, who noted many points of dissatisfaction concerning the veterinary services:

Q23: “*The veterinary services, they only vaccinate oxen and so every time they call for animal vaccination it is only for oxen. They have never talked about the dog, nor about dog vaccination. This means that we are not informed about this aspect of the problem*.” (IDI: DO, male, LO).

On human health side, the interviewees reported that rabies was not part of a national control program, nor does it appear in the monthly activity reports (in French: *rapport mensuel d'activité*, RMA) of health facilities. This means that rabies surveillance is currently not performed by the human health sector. In the FGDs, providers from the human health sector explained that this neglect is due to the lack of epidemiological information about rabies in humans, which would be important to apprehend the burden of the disease. Another reason for the neglect of rabies by the human health service is its zoonotic origin as a pharmacist explains. This is apparently leading to confusion about responsibilities between the sectors.

Q24: “*There is no rabies control program in Chad. Even in the RMA rabies is placed as ‘other case.' It is not written down as a case of rabies. But if a case of rabies is found, it is counted with the other diseases. This negligence comes from the fact that people do not understand the incidence and prevalence of this disease among the population*.” (Extract from FGD: CDMO, male, HL)

Q25: “*Rabies does not appear in the monthly activity report. Normally rabies should also be a disease under surveillance like the other diseases. But unfortunately, we don't see this disease in the monthly report. I also believe that this is a reason why health professionals sometimes forget about this disease. I also see that rabies is a hybrid disease between the human and animal medical professional. So that's why this disease is sometimes forgotten. […] But if it existed at the RMA level, I think that health professionals would have an eye for it*.” (Extract from FGD: HCM, male, OU)

#### Availability of PEP and animal rabies diagnosis

The health system in Chad is organized in a pyramid with three levels: the central level, the intermediate level and the peripheral level. The health center is the basic structure in the provision of care, and it is from the health center that the Minimum Package of Activities (MPA) is offered, consisting of preventive and curative activities. Bites cases are managed in these centers and should be covered by MPA. Health professionals explained that a few years ago, the Ministry of Health made rabies vaccines available to districts and health centers to treat bitten people free of charge, but for more than 3 years this free service has been suspended. This situation has led the managers of peripheral health structures to systematically refer bitten people to the larger cities like N'Djamena for PEP. In most human health structures, there are equipment shortcomings. The lack of a cold chain to store the vaccine was the major difficulty to supply vaccine to almost half of the facilities involved in the GAVI-project (authors own observation). This is also highlighted by the qualitative data.

Q26: “*Yes, we have problems with the cold chain. In our health centre, we don't have a cold chain, that was our great difficulty. We often call the District to send patients who are bitten by rabid dogs to be vaccinated*.” (IDI: HCM, male, OU)

Q27: “*Previously, it was difficult for us who are in the provinces […]. Because for every case of a bite, it was systematic, we were obliged to refer the patient to N'Djamena to get doses of vaccine and systematically, we killed the dogs. Whether they were domestic dogs or well-known dogs, even here, it was systematic, there was no observation period*.” (Extract from FGD: CDMO, male, CB)

The quote above also refers to the lack of access to animal rabies diagnosis leading to the negative practice to kill a dog after a bite already mentioned in the result section on the community level. According to the heads of the livestock services, reasons for inexistent follow-up of biting animals are the lack of resources and insufficient facilities. This is leading to the fact that some tasks under the responsibility of the veterinary services are actually performed by human health wokers, which seems to frustrate the animal health workers.

Q28: “*If we take Adré, there are more than 20 health centres, so with their own resources they [human health workers] are really helping us, whereas in the livestock sector there are only 4 veterinary posts. Even these veterinary posts lack the means to travel. Yes, especially in terms of resources, they have sacrificed themselves and sometimes [human health workers] replace us, they make observations, as if they were veterinarians and they take care of the bitten animal, for example*.” (Extract From FGD: CLS, male, OU)

Responses from participants from the human health sector underscore this lack of capacity of the animal health sector. Many health personnel think that veterinary services should be reinforced with facilities; equipment and training to be able to better do their work. One of the respondents even points out that this investment into better animal observation and rabies diagnosis will have a positive impact on the human health side through vaccine savings.

Q29: “*There are many bites, and we are not sure. We are limited by the means of diagnosis. It's difficult to put the dog under observation, it's difficult to make a biological diagnosis to confirm whether the dog that bit the person is sick or not*.” (Extract from FGD: CDMO, male, CB)

Q30: “*But if there are no trained people, even if there are buildings, we can't follow a dog. We treat people, so I think that these veterinary centres need to be provided with staff, enclosures and then we need to think about the animals' food. […] Especially the cost of the vaccine, we can't give a person who isn't sick three doses, whereas if we observe the dog and the dog isn't sick, we stop, that makes it possible to save on the vaccine*.” (Extract from FGD: CDMO, male, HL)

#### Attitudes and practices of service providers

Among the participants, the compliance of health personnel to PEP recommendations was found to be adequate, except for the non-use of RIG due to unavailability. Similar to the self-reported attitudes and practices by the community, this result also needs to be interpreted with the necessary caution. The participating human health and animal health providers have received training before the GAVI project implementation and their compliance to the PEP scheme (ESSEN or Thai Red Cross, excluding RIG) was followed up during the project. The interviews indicate that health personnel advices the community to wash the wound with soap and water after exposure before coming to the health facility. At a health center a wound is usually treated with antiseptics and then with anti-biotics and anti-tetanus vaccine. Providers also explain that the first treatment with rabies vaccine is adviced before any possible results from the animal investigation by veterinary personnel.

Q31: “*As soon as we receive a person bitten by a rabid animal, even if the animal is unknown, […] we first apply an anti-septic washing and vaccinate it. We inform our veterinary colleague, […] We have also made the population aware that as soon as a person has been bitten […], he must carefully wash the wound with soap and water before taking it to the health centre. At the health centre, we will carefully treat the wound, provide care and vaccination*.” (Extract from FGD: HCM, male, OU).

On the veterinary level results of our study show that some livestock technicians are reluctant to handle samples taken from the dog's body due to the religious taboo to handle a dogs carcass mentioned above (Q11). This constitutes a barrier to diagnosis even if resources are available.

Q32: “*For example, in Djarmaya, when there is a case, we automatically call the chief district medical officer and inform him of the veterinary agents, but they don't come. The one in Karal came twice, and then when we call him, he says that he is very far away and cannot come. The one from Pont Bélillé is often there, but his phone doesn't answer, they don't want to collaborate with us*.” (Extract from FGD: HCM, male, HL)

Q33: “*The difficulty is getting the dog's head to the laboratory for diagnosis, as you said earlier. It's a bit difficult as in Abdi where they are. Also here, cutting the dog's head, they are not ready to do that. Either, he cuts badly or he cut it yesterday and it's today that he shows up saying ‘I cut it yesterday' and he didn't even leave it in the fridge*.” (Extract from FGD: CVO, male, CB)

In regard to implementation of a OH approach, prior to the study the two sectors apparently operated independently, but the implementation of IBCM during the GAVI-project allowed for collaboration that was appreciated by both sectors as a favorable for rabies control. According to the providers, this collaboration allowed for the pooling of skills. However, in practice, difficulties were noted by the providers. Firstly, because of the very small number of veterinary structures compared to human health structures (see also Q35 above), which did not allow veterinary staff to collaborate effectively. Secondly, among the human health facilities, nearly half could not offer PEP, and victims were systematically registered and referred, because these facilities did not have a cold chain to store the vaccine (see also Q26 and Q27).

Q34: “*Before the project, everyone managed their own area: the veterinarians managed livestock and the health workers managed health, so we didn't really mix, but now I think we speak the same language*.” (IDI: HCM, male, LO)

Q35: “*Another difficulty in relation to care is the problem of collaboration between human health and veterinary staff. Here in our district, we only have one veterinary post in Krim-Krim, while at the human health level there are many HCs, so for the veterinary nurse to go round to put the dog under observation is a lot of trouble, so the work of the veterinarian with the community is a problem*.” (IDI: HCM, male, LO)

Q36: “*Inter-sectoral collaboration, it's an approach that is really commendable. It allows us to put together skills to fight against diseases. When one sector works in a closed way, it is difficult. Because as we have seen with rabies, clinically we can take care of the patient, but the diagnosis poses a problem. If there is no collaboration, it will be difficult to implement the diagnosis to allow for proper management. As we have begun, we will stay the course so that all diseases can be properly managed for the health of the population*.” (Extract from FGD: CDMO, male, LO)

Finally, the participants highlighted the need to integrate the community in the OH approach. This nicely closes the cycle from community to human and animal health institutions and back to the community.

Q46: “*But there is still a need to strengthen communication between the human health sector and animal health. In terms of the community component, we should also think about integrating community surveillance in the future, because this community surveillance will strengthen the identification of bite cases in households that can be referred to the health centers so that the patients can be treated*.” (Extract from FGD: HCM, male, CB)

Q47: “*What is important is to advocate, to raise awareness, so that the highest authorities can allow us to have an ideal framework that will bring us all together, so that we speak the same language and truly commit ourselves to fighting this disease. Indeed, […] the community aspect is very important. At this level, we must also take into consideration that in the future, we will have to sensitise the community to first of all know that the disease exists. […] We have to make them understand that if the animal is with them, the animal must be protected, the animal must be vaccinated*.” (Extract from FGD: CDMO, male, CB)

**Figure 2:**
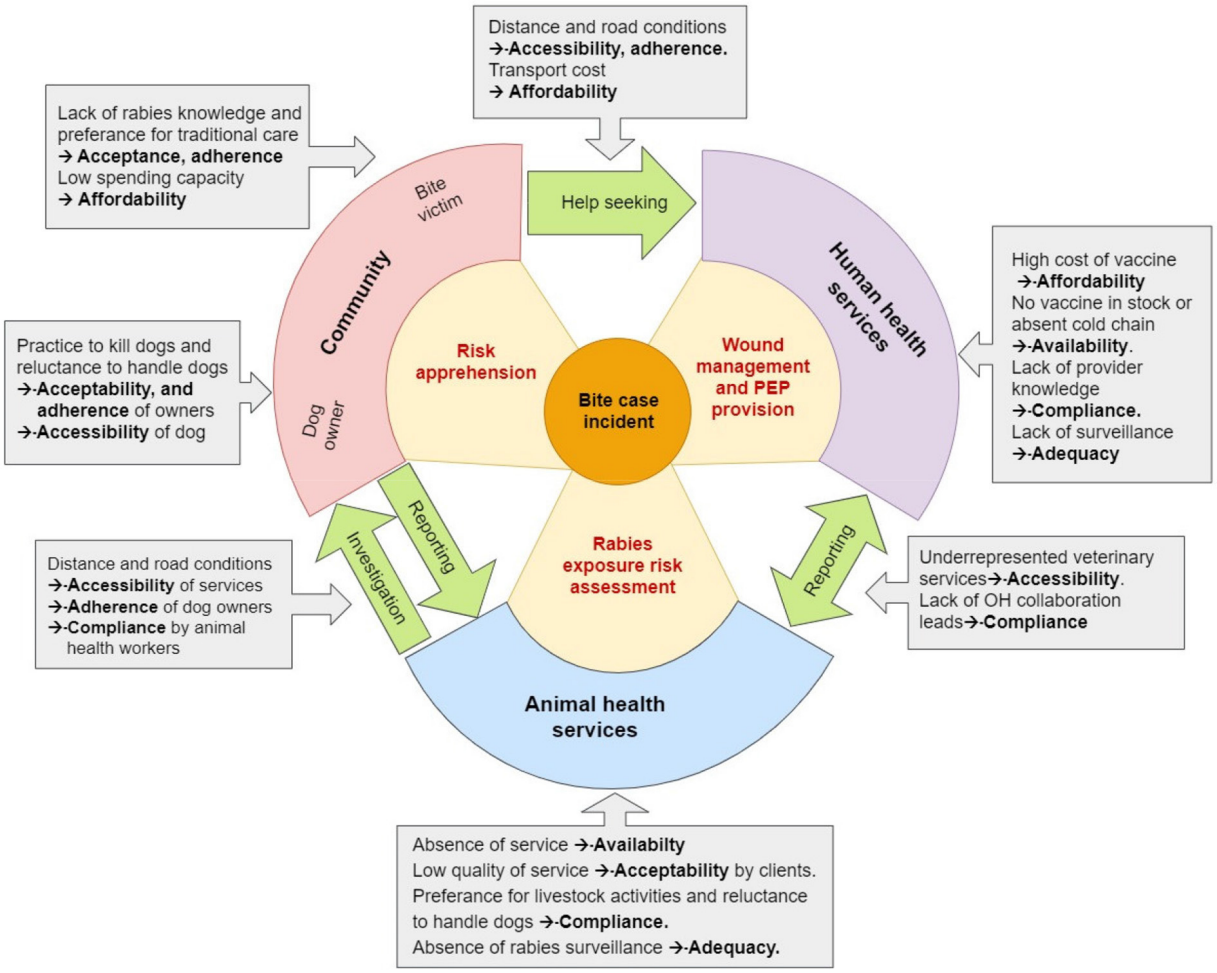
IBCM access cycle introduced in the introduction (Figure 1). This time the gray boxes highlight the barriers identified by level and linkage followed after an arrow by the corresponding access elements they influence.

To summarize the results we have adapted Figure 1 to show the challenges identified which influence on several access elements and on different levels of the access cycle (Figure 2).

## Discussion

This study was conducted with the aim of identifying the main factors that constitute bottlenecks to rabies control in Chad following the OH approach. The results reveal numerous obstacles on community and institutional level, many of which are interdependent and therefore influence several access elements at a time. To provide a graphic overview of the study results we adapted Figure 1 to show the identified obstacles by level of actors and access element. The factors identified cannot be considered exhaustive and are only to a limited extent representative for the national Chadian context. This is because the recruitment of participants did not take into account criteria of gender, socio-economic status, cultural or educational background of community or institutional representatives. Access differences based on these criteria certainly exist, but could not be investigated through our study due to the limited methodological design. The results also need to be interpreted in the context of the GAVI-project that has certainly increased knowledge of rabies prevention and control on both the community and the service provider level during its 2 years period. Despite these major limitations we think that the results are valuable to give a rough overview of the major access barriers to be addressed in Chad for a future implementation of IBCM. The results also help to find explanations for the quantitative findings related to weak PEP access and animal bite reporting despite the efforts of the GAVI-project to increase PEP and animal rabies diagnosis availability.

Not surprisingly, affordability of human rabies vaccine is among the main issue determining PEP access. People and providers are unanimous on this point: PEP is extremely expensive. During the GAVI project human vaccine for PEP was provided free of charge in health centers and hospitals participating in the bite survey, provided they could insure cold chain. The cost of human RIG could not be covered by the project and equine RIG, which is less expensive is not licensed for use in Chad. Outside of the project activities, the price of human vaccine was estimated by the respondents at 12,000 CFA francs (18 EUR) per dose and the overall cost of PEP at 50,000 CFA francs (76 EUR), excluding the cost of consultations and wound treatment. This amount is out of reach for poor households in the country. Obstacles to treatment in health facilities resulting from the high cost are a consequence of poverty as a study in Cameroon has highlighted (26). Chad, although an oil country, is a poor country with about 47% of the population living on <US$2 per day (http://data.worldbank.org/pays/Tchad). Affordability of veterinary services was not investigated by this study. For both PEP access and animal investigation, long distances to facilities, transport difficulties related to poor road infrastructure and adverse climatic conditions during some time of the year add to the cost of the actual service. The same factors also explain another major obstacle, which is geographical accessibility of human and animal health facilities. For access to PEP this problem is exacerbated by the low availability of human vaccine in local health facilities. Our results illustrate previous quantitative findings on affordability and geographical accessibility of PEP (18, 24). The average distance patients travel to a health center, for example, is 16 km and to a hospital is 62 km (information from Ministry of Health health statistics, https://reliefweb.int/report/chad/tchad-annuaire-des-statistiques-sanitaires-tome-31-me-dition-ann-e-2017). Geographical distance was also found to be a major determinant of access to PEP in other countries such as India (27) and Tanzania (28). Unable to access primary health facilities or secondary facilities they are referred to, poor communities turn to other therapeutic methods such as traditional treatment. Health providers therefore stated quite clearly: If nothing is done about the cost and availability of the vaccine, people will continue to die of rabies. The solution to this problem would be government investment in human vaccines for PEP to ensure availability and accessibility to prevent deaths. The new GAVI investment strategy could support governments of eligible countries in the free provision of human rabies vaccine for PEP, provided that a country has adopted a national rabies action plan (6). The implementation of this strategy was unfortunately delayed due to the COVID-19 pandemic. Moreover, Chad currently has no rabies action plan validated on the ministerial level.

Depending on the schedule, a course of PEP includes between three and five visits to a health center. Therefore, affordability, accessibility and availability are all impacting on patience adherence as our qualitative data clearly illustrate. In Tanzania, too, the risk of non-adherence PEP was high for people living far from health facilities (28). On the other hand geographical accessibility of communities is mentioned by service providers as an obstacle to carrying out their work, such as sample collection for rabies diagnosis. It also hampers the outreach and sensitization strategy of the human health sector to cover very remote populations and therefore adequacy.

Another constraint to accessibility of rabies care identified in this study is the decision-making process. The results of the interviews highlighted the parental influences and power differentials that condition health seeking behavior (Q18). Due to the high costs of PEP or the distance to a health facility, decision-making constraints often have negative implications for health care, especially when the victim is a child or a woman. In general, when it comes to health in a household or family, the head of the household decides and his or her agreement is a prerequisite in this process. Similar parental dynamics have been observed in Tanzania (29). However, to investigate on the dynamics of gender and age dependent access barriers in Chad a further in depth study would be needed.

The interviews reveal that traditional care is strongly used by Chadian communities. In the quantitative baseline study, 24% of bite victims consulted traditional healers (18), and the qualitative results also confirm this trend. The follow-up of the therapeutic itinerary of bite victims in the study conducted by Mindekem et al. (24) identified the same problem. In some cases, traditional care providers even seem to discourage patients from seeking PEP (Q9). According to health service providers, this attitude of local healers has caused the death of several victims during the GAVI-project, which shows that even if solutions to geographical accessibility, availability and affordability can be found, issues concerning acceptability of PEP by some people will remain. The importance attached to traditional care gives providers a mere parallel role in the provision of care for a disease as severe and fatal as rabies. However, it seems clear that difficulties related to cost, availability or geographical inaccessibility is the main driver for bite victims to resort to other therapeutic alternatives at the expense of proper post-bite medical care.

Health seeking after a bite is also strongly influenced by risk perception and in consequence by the level of knowledge a community has about the severity of rabies to adequately realize the importance of accessing PEP. Although most participants from the community knew that rabies is a disease transmitted from dog to dog or from dog to humans by bite, knowledge of other vector animals or of the paralytic form or rabies seems to be lacking. Moreover, many victims seem to neglect minor wounds or scratches (Q19) and think that the risk is mitigated when the wound has healed (Q4). Since all participants in our study had sought help at health facilities, we were unable to assess the impact on knowledge on health seeking in more detail. However, the sad story of the death of a child that a nurse shared during one of the interviews (Q8) indicates that the lack of awareness of the lethal nature of rabies might have an influence on the efforts undertaken by victims or parents to access PEP. Interestingly knowledge level was not significantly linked to preference for either medical or traditional care in Cameroon (26), but results from Kenya show that knowledge of rabies was the main driver in seeking medical attention after a bite (30).

Another factor influencing the motivation to access a service is a client's perception of the quality of this respective service. Quality of services is besides availability also impacted by adequacy and compliance. The FGDs and individual interviews denounce especially that the veterinary services that would be responsible to provide animal vaccination, observation of biting animals and rabies diagnosis are not readily available and accessible. Participants reported a lack or inadequacy of technical and logistical facilities and trained rabies personnel, which limits the performance of this sector. Similarly to PEP, animal observation includes several visits and therefore geographical accessibility is also impacting on adherence (if the dog owner needs to bring his animal to the veterinary facility) or compliance (if the animal health worker needs to visit a dog owner). The challenges for rabies control related to availability and poor quality of veterinary services was also encountered during the extended surveillance study of the GAVI-project (21) and are also reported from other rabies endemic countries, for example Ghana (31). The qualitative findings of this study confirm results from a knowledge, attitudes and practices survey done on the occasion of the training workshops at the beginning of the GAVI-project. This study revealed deficits in knowledge and practice (KAP) for rabies service delivery in both human and animal health personnel (23). Even if there are facilities and trained personnel, rabies control tasks seem to be neglected in favor of other, more lucrative activities. Adding to this are religious barriers to work with dogs, as our qualitative data clearly illustrate. Especially animal health workers with a Muslim religious background express their reluctance to collect and diagnose samples from dogs. They feel that their families and close relatives would not eat off the same plate with them if people were to learn that they had handled a dog carcass. This could be an explanation for the very few samples received from the northern study area during the GAVI project, indeed one of the decentralized diagnostic units established in the Ouaddaï province only sent a donkey sample to the central rabies laboratory in N'Djaména (21). This lack of compliance of animal health workers further undermines veterinary services and on the community level the same hesitance to handle dogs constitutes a barrier to dog vaccination (23). On the other hand, in the southern regions of the country, dominated by a Christian religious background, dog meat is a source of protein for some communities. This practice results in the abusive slaughter of dogs after a bite case, which seriously compromises dog observation and sample taking. Again, our qualitative data confirms quantitative findings on this practice published in Naïssengar et al. (21). Finally, our study shows a lack of reinforcement of the law on compulsory reporting of rabies suspicious animals. The community and the animal health services seem to blame themselves vice versa for this shortcoming. However, it should be a government's responsibility to adequately inform the public about laws and provide the means to the respective services to reinforce these laws. Because of this negligence on governance level, animal health services are not really contacted by dog owners after a bite, because they are not aware that they are obliged by law to do so and they do not trust this service to be able to respond to their demand.

A similar neglect is observed on the human health side. Providers deplore that nothing has been done about rabies since the adoption of the law mentioned above: there is no national health action plan on rabies and the disease is not included in the list of diseases under surveillance in humans. The monthly activity reports of health facilities mentioned by participants (Q24 and Q25) exemplify the lack of data. For some providers, this lack of data explains why rabies remains neglected. A study in Côte d'Ivoire documented the very positive effect that increased efforts on human rabies surveillance can have on the epidemiological data situation (32). Another possible explanation is that human health professionals think it is the responsibility of the animal health sector to control this disease since rabies is of animal origin. However, it would be clearly the responsibility of the human health services to provide PEP to potentially rabies exposed people. Theoretically human rabies vaccine should be included in the MPA of health facilities, but in practice, availability and affordability of this life saving product constitute a huge barrier to access. Human health officials explained that in the past few years, the free human rabies vaccine supply through the ministry of public health has been suspended for reasons that are unknown. This suspension is having a very detrimental impact on PEP coverage with only not even 10% of bite victims accessing PEP in Chad as quantitative results from the GAVI-project household survey show (18). Compliance of human health workers to WHO recommended PEP schemes could not be assessed by this study, but the afor mentioned KAP study (23) and the PEP follow-up data on the health facility level (18) during the GAVI-project found deficits in provider knowledge and compliance.

On the positive side, providers show committement to the strategy of collaboration between the human and animal health sectors. This confirms findings of the KAP survey back in 2016, where both human and veterinary workers well identified the need and value of better collaboration (23). Nevertheless, the interviews also revealed that practice lags behind the positive intentions to collaborate. A similar lack of practical implementation of intersectoral collaboration has been reported from Gomma in the South West District of Ethiopia (33). Challenges for this collaboration result mostly from lack of capacities on the animal health side. This is illustrated by the fact that the number of veterinary structures and staff is numerically much lower than on the human health side. At the regional level, the ratio is about four health centers for one veterinary post. This means that animal health facilities are even harder to access than human health facilities and on the other hand animal health workers often have to travel long distances for animal investigation. Besides the obvious need for capacity strengthening on facility level and training of personnel, ways to make rabies control activities more profitable for the animal health sector would need to be found to provide sufficient incentive for OH collaboration. For example, samples collected in the study areas by designated animal health personnel were rewarded with 5,000 FCFA (about 9 USD), which was certainly one of the main drivers for reporting and testing suspect cases (21). Another option would be to quantify the economic losses due to rabies in the livestock sector as recently done by a study in Ethiopia (34). This would show the benefit of investment into rabies control for this important economic sector in Chad.

Regarding the community level, we identified limitations of awareness to inform and influence individual and community habits about rabies that might hamper success of OH interventions. Although the knowledge of the main transmission pathway from dogs to human is known, there are deficits in risk perception and help seeking in human health facilities after a bite. This alone constitutes a limiting factor to IBCM, but the biggest challenge are cultural practices regarding the reluctance of dog handling on the one hand side and consumption of dog meat on the other hand. Both practices limit access to dog investigation and hence efficient IBCM. Lack of community awareness that could negatively affect appropriate rabies prevention measures are also reported from other African countries, for example the Democratic Republic of Congo (35). Interpersonal communication (with rabies images) should help reduce prejudice and could have positive influence on attitudes and practices toward the use of medical and veterinary services in case of a bite. To this end, it would be interesting to develop integrated communication systems using community relays, community-based associations and religious channels. Since traditional healers are frequently contacted after a bite, they would need to be included in a sensitization and awareness campaign.

## Conclusion

Rabies prevention and control faces enormous challenges in Chad. These challenges are multilayered, meaning encountered on community, human health and animal health service level. They are closely interrelated and of various nature including economic, socio-cultural and organizational. Looking at all these obstacles one could become easily discouraged, but the interrelatedness of the access elements and factors affecting them is also a chance: minimizing or eliminating one barrier will have positive effects on several others. For the conclusion of our study, we would like to highlight two leverages by level of actors that we feel would be the most urgent to be addressed and also highly impactful to improve IBCM access. Firstly, on the community level public awareness campaigns will be an activity with the strong potential to overcome barriers related to lack of knowledge and erroneous attitudes and practices.

On the human health level, the most urgent task would be to decrease the price of human vaccine for PEP or provide it entirely free of charge and to start reporting human rabies deaths. On the animal health side, it would be crucial to provide a financial stimulus for rabies control activities and increase government investment in the veterinary sector. Doing so would decrease cost in the human health sector and potentially has economic benefits for the livestock sector. Finally, from the experience of our work we think that joint training workshops with human health and animal health workers fuel intersectoral collaboration besides the positive effect on provider knowledge and compliance.

As we write this publication an interministerial OH platform is being established in Chad. We strongly hope that one of the first actions of this platform will be the adoption of a national rabies action plan similar to those existing already in other African countries (36). Besides securing free human rabies vaccine through the GAVI-investment strategy this would be a strong sign for commitment to the global agenda toward zero human deaths from dog mediated rabies by 2030 (7).

## Data availability statement

The raw data supporting the conclusions of this article will be made available by the authors, without undue reservation.

## Ethics statement

The studies involving human participants were reviewed and approved by Ethics Committee of North Western and Central Switzerland (EKNZ). Written informed consent for participation was not required for this study in accordance with the national legislation and the institutional requirements.

## Author contributions

AM: health geographer, study design, data collection, analysis, and manuscript writing. ML: veterinarian and rabies expert, study coordination, and manuscript writing support. KH-T: sociologist and manuscript proofreading and revision. JZ: veterinarian, One Health expert, and study PI. MD: One Health expert, support for analysis approach, and manuscript revision. NS: sociologist, methodological, and manuscript writing support. NM: sociologist, study co-design, data collection, and analysis support. All authors contributed to the article and approved the submitted version.

## Funding

This research is part of a project conducted under the DELTAS Africa Initiative [Afrique One-ASPIRE /DEL-15-008] and the Global Vaccine Alliance (GAVI) learning agenda (Exhibit A-3 PP46311015A3C). Africa One-ASPIRE was funded by a consortium of donors including the African Academy of Sciences (AAS), the Alliance for Accelerated Scientific Excellence in Africa (AESA), the New Partnership for Africa's Development (NEPAD) Planning and Coordinating Agency, the Wellcome Trust [107753/A/15/Z] and the UK government.

## Conflict of interest

The authors declare that the research was conducted in the absence of any commercial or financial relationships that could be construed as a potential conflict of interest.

## Publisher's note

All claims expressed in this article are solely those of the authors and do not necessarily represent those of their affiliated organizations, or those of the publisher, the editors and the reviewers. Any product that may be evaluated in this article, or claim that may be made by its manufacturer, is not guaranteed or endorsed by the publisher.
