# Supplementary material for: “When a dog bites someone”: Community and service provider dynamics influencing access to integrated bite case management in Chad
Source: Front Vet Sci. 2022 Oct 10;9:866106. doi: 10.3389/fvets.2022.866106 (PMC9588941; doi:10.3389/fvets.2022.866106)
Supplement: Supplementary file 1 [file Data_Sheet_1.docx]

**Supplementary document Nr.1**

**Discussion points addressed during Focus Groups and interview questions by participant group**

**Points discussed during focus groups with dog owners and bite victims**

1. How do you describe the dog (*probe what the dog represents, how does your community perceive the dog*)?
   1. The advantages of owning a dog
   2. The disadvantages of owning a dog
   3. The risks of owning a dog
2. In your community, what are the specific perceptions of responsible dog ownership?
   1. What should a dog owner do?
   2. What should a dog owner not or cannot do?
3. In your community, what actions are taken in the event of a dog bite from a suspected rabid dog?
   1. Who makes the decision?
   2. What should be done with the dog?
   3. What should be done with the victim?
4. Searching for and seeking care for rabies in your community
   1. What are the possibilities for care in case of a bite?
   2. What are the underlying motivations for using medical or traditional services?

**Points discussed during focus groups with health centre managers (HCM) and chief veterinary officers (CVO):**

1. What services does your centre offer in the field of rabies (human and animal health)?
2. What are the services most requested by bitten people or owners whose dogs have bitten (human and animal health)?
3. What are the least used services and why?
4. In the communities served by your centre, are there any factors that explain why bitten people refuse to come to a health or veterinary centre in case of bites (cultural, economic, religious factors, marital status)?
5. In the communities served by your centre, are there any factors that explain why bitten people would decide to consult traditional healers (cultural, economic, religious factors, marital status)?
6. How are the services organised in the different health sectors (opening hours, type of staff, procedure)?
7. To what extent is the demand for PEP consistent with the needs and expectations of the population?
8. Availability of services?
9. Geographical accessibility/setting?
10. Waiting time?
11. Competence of providers
12. Accessibility to information sources?
13. As service providers, how do you perceive the quality of services offered in regard to rabies prevention and control?
14. How do you feel about the state of your facilities to provide rabies prevention and control?
15. How did the health centres communicate or collaborate with the veterinary services during the project period and what are the elements of this collaboration?
16. How did the decentralized diagnostic units established help you?
17. Rabies is a disease that requires the collaboration of both sectors for effective control, what can be done to improve this collaboration?
18. Suggestions and solutions
    1. What do you expect from the Ministry of Health and Livestock in terms of access to care for human and animal vaccines?
    2. If you were told to put in place a strategy to ensure that humans and dogs have access to vaccination and care, what would you do?

**Points discussed during focus groups with chief district medical officers (CDMO) or district hospital chiefs (DHC) and chiefs of livestock sectors (CLS):**

1. Did the results of the Gavi project surprise you?
2. In your district or sector what are the rabies needs?
3. How did you identify these needs?
4. Is there an action plan for the control of rabies?
5. What actions have been taken regarding the control and prevention of rabies?

- In the human health sector?
- In the animal health sector?

1. What do you think of the animal rabies diagnostic units established by the project?
2. Is the human or animal rabies vaccine taken into account in the package of free medical products?
3. If so, what exactly is the situation with regard to free human and animal rabies vaccine?
4. Why do you think rabies is still a neglected disease, especially since it is so lethal?
5. What do you think of the strategy of intersectoral collaboration and particularly between human and animal health during the GAVI project period for the fight against this disease?

**Interview questions for dog owners (DO):**

1. **Practices and perception around the dog and knowledge of rabies**
   1. Have you or any member of your household been bitten by a dog in the last 12 months?
   2. What risks might a dog owner be exposed to?
   3. What responsibilities do you think a dog owner should take towards their pet?
   4. What should you do if you are bitten by a dog?
   5. Do you know what diseases a dog can transmit to humans?
   6. Have you heard of rabies?
   7. Have you ever seen a rabid animal and if so, what are the signs?
   8. Have you ever had a rabid animal in your household or family if so, how did it change your perception of the disease?
2. **Access to and use of veterinary services for surveillance and observation**
   1. Is there a veterinary station in your area?
   2. What services have you contacted at the veterinarian's office and how do you rate the services offered by this office?
   3. Are you aware of the practice of putting an animal under observation after a bite? (If no, please explain)
   4. Would you be willing to implement this surveillance and prevention measure after your animal has bitten someone? What would be the obstacles to a clean observation (chaining or locking up the dog for 10 days)?
   5. Are you aware of the possibility of rabies diagnosis after the death of an animal?
   6. Would you be willing to use this diagnostic service and if so, what do you think would be the positive aspects of the test and of animal surveillance in general?

**Interview questions for bite victims (BV):**

1. **Practices and perceptions around dogs and knowledge of rabies**
   1. Have you or any member of your household been bitten by a dog in the last 12 months?
   2. Do you think the bite could have been prevented by more appropriate behaviour towards the dog?
   3. What risks might the victim be exposed to?
   4. What should you do if you are bitten by a dog?
   5. Do you know what diseases a dog can transmit to humans?
   6. Have you heard of rabies?
   7. Have you ever seen a rabid animal and if so, what are the signs?
   8. Have you ever seen a person with rabies and if so, what are the signs?
2. **Access to and use of human health services**
   1. Is there a health centre in your locality?
   2. How do you rate the services offered by this centre?
   3. What difficulties do you encounter in relation to PEP and care?
   4. What are the constraints in your environment that prevent access to rabies vaccination (PEP)?
   5. Is there other care available for rabies treatment in your locality and if yes what does it consist of?
   6. Is there a veterinary post in your community?
   7. How do you rate the services offered by this post?
   8. Do you have anything else to add?

**Interview guide for health centre managers (HCM) and chief veterinary officers (CVO):**

1. **Use of services**
2. What services does your center offer in the area of veterinary care or dog bites?
3. Are these services used?
4. Which rabies services are most frequently used?
   1. Which rabies services are least used?
   2. Why are these services used less?
   3. In the communities covered by your centre, what are the factors that prevent access to rabies care?
5. **Evaluation of the quality of services**
   1. How is your service organised in the area of access to bite case management? (Schedule, type of staff, procedure)
   2. How well does this service match the needs and expectations of the population?
      1. Availability of services
      2. Geographical accessibility
      3. Financial accessibility/cost of services: dosage, total cost of treatment.
      4. Competence of providers, how PEP is administered, what protocols are frequently used?
      5. Accessibility to information
   3. As a provider, how do you perceive the quality of care you offer?
   4. What difficulties do you encounter in dealing with rabies-suspect animal bites?
6. **Communication support**
   1. How does the centre communicate with the population about access to care and dog observation? (On what occasion, the message and who does it)
   2. Does your service have the tools (leaflets, image box...) to support communication with the population?
   3. Do you think that the population perceives the message?
7. **Conclusion**
   1. If you were asked to put in place a communication strategy to increase the population's use of PEPs and vaccination of their dogs, what would you do?
   2. How would you communicate more effectively with the public about access to care and dog vaccination in your community?
   3. Do you have anything else to add?
